# Time for a diagnostic sea-change: Rethinking neglected tropical disease diagnostics to achieve elimination

**DOI:** 10.1371/journal.pntd.0008933

**Published:** 2020-12-31

**Authors:** Katherine Gass

**Affiliations:** Task Force for Global Health, Decatur, Georgia, United States of America; Institute of Continuing Medical Education of Ioannina, GREECE

## Introduction

Never before has the importance of good diagnostic tests received such global attention. With the Coronavirus Disease 2019 (COVID-19) pandemic, sensitivity and specificity have become household terms. For neglected tropical diseases (NTDs), the need for good diagnostics is nothing new, but has grown increasingly acute in recent years. While today’s diagnostic tools have been sufficient for endemicity mapping and monitoring the progress of mass drug administration (MDA), they are largely insufficient for making stop treatment and surveillance decisions.

A diagnostic sea change is required to develop new tests capable of taking NTD programs to the finish line of elimination. This starts with recognition that the testing needs for NTDs are unique. Unlike malaria, tuberculosis, or HIV, the preventative chemotherapy (PC)–treated NTDs are largely asymptomatic, and consequently, tests are rarely used to make individual diagnoses. Instead, NTD programs rely on tests to make good public health decisions. These decisions are made by applying diagnostic tests in conjunction with World Health Organization (WHO)–approved monitoring and evaluation (M&E) tools, which typically take the form of population-based surveys. Accurate program decisions can only be made with tests that are fit for purpose. Deploying such tests will require the NTD community to make 4 important changes in the way diagnostic tests are developed and applied.

## Recognize that specificity determines a test’s utility for endgame decision-making

Moving from individual to population-based decision-making requires that one think differently about sensitivity and specificity, as these metrics in isolation will not provide sufficient information on the risk of making an incorrect decision. For the PC NTDs, stop MDA decisions are made by comparing the observed survey prevalence against a target threshold (e.g., <1% prevalence of soil-transmitted helminths). The observed prevalence is dependent on the number of individuals testing positive, which will include true positives and those testing positive falsely. The probability that an observed positive result is indicative of a true positive is characterized by the positive predictive value (PPV). Similarly, the negative predictive value (NPV) is the probability that someone testing negative is truly negative. It is important to note that while disease prevalence has no impact on the sensitivity or specificity of a test, it does impact the PPV and NPV. As the prevalence of disease decreases, the PPV will approach 0, while the NPV will approach 1. The definitions for PPV and NPV are derived using Bayes theorem for calculating conditional probability and are shown in Eqs 1 and 2 below [1].

$$PPV=\frac{sensitivity\;\times \;prevalence}{sensitivity\;\times \;prevalence+(1-specificity)\;\times \;(1-prevalence)}$$ (1)

$$NPV=\frac{specificity\;\times \;(1-prevalence)}{\left(1-sensitivity)\times \;prevalence+specificity\;\times \;(1-prevalence\right)}$$ (2)

While a diminishing PPV is important to appreciate when interpreting test results in the context of disease elimination, this assumes that sensitivity and specificity are fixed; however, when developing a new test, sensitivity and specificity are mutable and often the targets to be optimized. If the goal is to end up with a new test that is explicitly designed to perform well in low-prevalence settings, what values of sensitivity and specificity should go into the target product profile (TPP)?

To answer this question, it is helpful to understand the interplay between sensitivity, specificity, PPV, NPV, and prevalence. Consider the 3 low prevalence settings of 1%, 2%, and 5% infection (note that each of these represents a threshold currently used to make stop MDA decisions by 1 or more of the PC NTDs). For each prevalence setting, 2 simulations are run: (1) the sensitivity is fixed at 99% while the specificity varies from 80% to 100%; and (2) the specificity is fixed at 99%, and the sensitivity varies from 80% to 100%. For both sets of simulations, the PPV and NPV are calculated for each sensitivity–specificity–prevalence trio.

**Fig 1:**
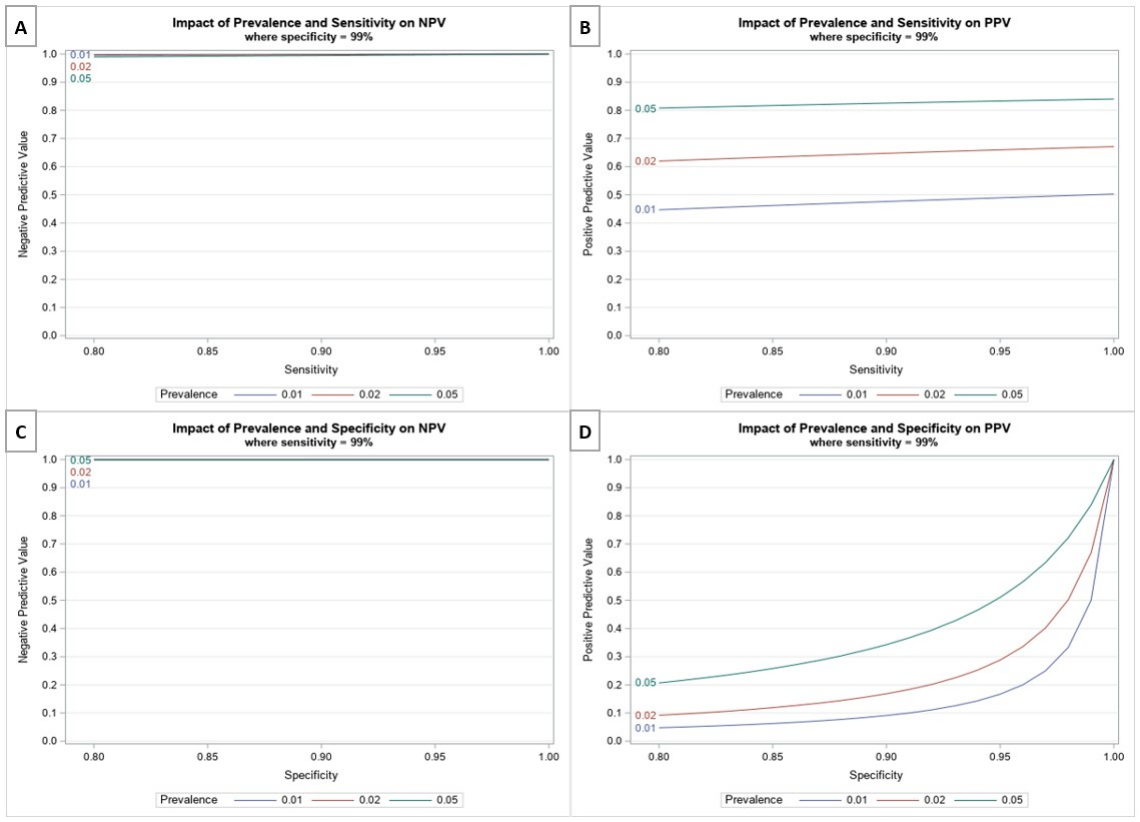
Simulating the impact that changes in sensitivity and specificity have on the PPV and NPV for 3 prevalence thresholds: 1%, 2%, and 5%. (A and B) The impact that varying the sensitivity from 80% to 100% has on the NPV and PPV, respectively, holding specificity constant at 99%. (C and D) The impact that varying specificity (from 80% to 100%) has on the NPV and PPV, respectively, holding sensitivity constant at 99%. NPV, negative predictive value; PPV, positive predictive value.

One of the first things to notice when looking at Fig 1 is the apparent lack of impact that variations in either sensitivity or specificity have on the NPV (Fig 1A and 1C). When the prevalence of infection is low, the probability that someone testing negative is truly negative remains near 100%, even if the sensitivity or specificity is around 80%. Shifting to the PPV, if specificity is held constant at 99%, increasing the sensitivity from 80% to 100% has very little impact on the PPV, as evidenced by the flat slopes in panel B. When the prevalence of infection is 1%, the PPV remains around 50%, even if the test is 100% sensitive (Fig 1B). Conversely, if sensitivity is held constant (Fig 1D), increasing specificity has a dramatic impact on the PPV; increasing specificity by 20 percentage points (from 80% to 100%) increases the PPV by 5- to 10-fold. And yet, even with a test that is 98% specific, if the prevalence of infection is 2%, the results in Fig 1D indicate that nearly half of all positive tests would still be false.

A low PPV is problematic for NTD programs. All the PC NTDs currently base stop treatment decisions on either the number or proportion of positive tests observed. If a significant proportion of these positive tests are false positives, the NTD program may erroneously continue treating long after elimination has been reached. As Fig 1 demonstrates, in low prevalence settings, specificity drives the PPV of a test, while sensitivity has little impact on test interpretation. It may seem counterintuitive that sensitivity is less important when it comes to the end stages of a program; the common belief is that high sensitivity is needed to find the proverbial needle in a haystack. However, if a positive test is more likely to be false positive than true, a program conducting posttreatment surveillance will end up chasing more shadows than true signals, wasting valuable resources and time.

## Consider the M&E tool when developing new tests

Given the impact of specificity on test interpretation in low prevalence settings, it is important to consider the implications of applying a new test in the context of an M&E tool, such as a stop MDA survey. In particular, it is crucial to understand how errors in test performance (e.g., false positives or false negatives) get propagated when applied across a sample of individuals and the likelihood that these errors may lead to a false program decision.

Consider a new diagnostic test that is 99% specific. The probability that 1 uninfected person tests positive (falsely) is 1% (1 minus the specificity). Now, suppose one were to apply this same new test in the context of the lymphatic filariasis (LF) Transmission Assessment Survey (TAS), a population-based cluster survey used by programs to determine if it is safe to stop MDA with minimal risk of recrudescence [2]. A typical sample size for the TAS is 1,692 children [3]. If all 1,692 children are truly uninfected, the probability that they all test negative by the new test is 4.1 × 10^−8^ (0.99^1,692^). But more importantly, the expected number of false positives one would observe from such a survey is 17 (0.01 × 1692 = 16.92). This is of consequence to the LF program because the critical cutoff for continuing MDA is observing more than 20 positive tests. The binomial distribution can be used to calculate the likelihood of observing *x* positive results, given *n* people are tested with diagnostic that is *Se* sensitive and *Sp* specific and a disease prevalence of *p* as follows:

$$P(x)=\frac{n!}{(n-x)!x!}[(Se*p)+(1-Sp)*(1-p){]}^{x}*[(1-Se)*p+Sp*(1-p){]}^{n-x}$$

Note that in the equation above, [(*Se* * *p*) + (1—*Sp*) * (1—*p*)] is the probability that someone tests positive, while [(1—*Se*) * *p* + *Sp* * (1—*p*)] is the probability that someone tests negative. With a test that is 99% specific, the probability of exceeding the TAS critical cutoff, meaning observing >20 false positive tests out of 1,692 children who are all truly negative is 18.8%. If the true prevalence in the sampled population were 0.5% (well below the 2% prevalence cutoff for a TAS survey), then the chance of exceeding the critical cutoff and mistakenly continuing MDA is 83.1% (see S1 Text for R code). This type of test performance is a deal breaker for programs trying to reach elimination.

Experts charged with developing TPPs for new tests should consider the implications of different sensitivity and specificity values in the context of existing program tools. The sensitivity and specificity criteria set forth in a TPP should represent values that will result in sufficient decision-making performance within the applicable M&E survey.

## Be willing to move away from the single-test paradigm

Should diagnostic developers strive for creating perfect tests? No. Not only is 100% accuracy unachievable, but it is also unnecessary. Shifting from a single-test to a multi-test paradigm reduces the pressure to develop a single “perfect” test and may lead to a net specificity that is greater than either single test.

When it comes to combining tests, 1 option is to do so serially, where 1 test precedes the other. Serial testing is a common diagnostic tool used in a variety of public health contexts, most notably HIV [4,5]. A highly sensitive test is typically performed first, to rule in all potentially infected individuals, which is then followed by a highly specific second test, to confirm those that are truly infected. An important feature of this strategy is that the second test is reserved for the subset of individuals testing positive by the first test. Consequently, it may be acceptable for the second test to be a more expensive or labor-intensive laboratory assay (e.g., PCR) if the modest investment increase leads to better program decisions. When combining 2 tests (A and B) in this manner, the net sensitivity and specificity can be calculated as follows:

$$Sensitivity=A_{\text{sensitivity}}\;\text{x}\;B_{\text{sensitivity}}$$

$$Specificity=A_{\text{specificity}}+(1–{\text{A}}_{\text{specificity}})\;\text{x}\;{\text{B}}_{\text{specificity}}$$

To illustrate how such an approach can result in a net gain of specificity, consider test A that is 95% sensitive and 85% specific and test B that is 80% sensitive and 99% specific. Assuming the 2 tests are independent, the net sensitivity is 76% (0.95 × 0.8) and the net specificity is 99.85% (0.85 + 0.15 × 0.99).

An alternative to serial testing is parallel testing. With parallel testing, everyone is tested multiple times. This may take the form of independent tests, or a single test with 2 or more markers, such as the dual-antigen rapid diagnostic test used in malaria [6,7]. Because everyone receives both tests, an appealing aspect of this approach is that the results may be interpreted to maximize net sensitivity or net specificity as follows, assuming both tests or markers are independent:

- “Maximizing sensitivity”—if either test or marker is positive (A “or” B), then an individual is considered positive [8].
  $$Sensitivity=A_{\text{sensitivity}}+B_{\text{sensitivity}}–(A_{\text{sensitivity}}\;\text{x}\;B_{\text{sensitivity}})$$
  $$Specificity=A_{\text{specificity}}\;\text{x}\;B_{\text{specificity}}$$
- “Maximizing specificity”—both tests or markers must be positive (A “and” B) for an individual to be considered a true positive [8].
  $$Sensitivity=A_{\text{sensitivity}}\;\text{x}\;B_{\text{sensitivity}}$$
  $$Specificity=A_{\text{specificity}}+B_{\text{specificity}}–(A_{\text{specificity}}\;\text{x}\;B_{\text{specificity}})$$

To illustrate how the interpretation of parallel testing can lead to a net increase in either sensitivity or specificity, consider a lateral flow dual-band rapid diagnostic test, where the sensitivity and specificity of marker A is 95% and 85%, respectively, and the sensitivity and specificity of marker B is 80% and 99%, respectively. When the presence of either marker is interpreted as a positive result, the net sensitivity is 99% (0.95 + 0.8 − 0.95 × 0.8) and the net specificity is 84.15% (0.85 × 0.99). When both makers are required to be present for an individual to test positive, the net sensitivity is 76% (0.95 × 0.8) and the net specificity is 99.85% (0.85 + 0.99 − 0.85 × 0.99). Consequently, one can envision a programmatic scenario where a single dual-band test could serve 2 programmatic purposes: as a highly sensitive tool for routine monitoring and a highly specific tool to demonstrate absence of infection.

It is important to emphasize that these formulas for calculating the sensitivity and specificity of test combinations rely on the assumption that the test results are independent. When 2 tests measure a similar biological process (e.g., the presence of antifilarial antibodies), test results are likely to be dependent, conditional on the person’s disease status [9]. The presence of conditional dependence between tests means the net gains in sensitivity or specificity are likely to be reduced.

## Adapt M&E decision rules to reflect test performance

Regardless of what test, or combination of tests, is ultimately used for program decision-making, no testing strategy will be infallible 100% of the time. While test imperfections are no surprise, historically NTD programs have failed to account for test performance in the design and interpretation of M&E surveys. Unfortunately, this oversight may lead to an incorrect interpretation of M&E survey results. When epidemiologists design a survey to measure a threshold, they typically consider 4 different parameters: the critical cutoff, sample size, probability of type 1 error (e.g., the risk of falsely rejecting the null hypothesis), and probability of type 2 error (e.g., the failure to reject the null hypothesis when the alternative is true). In the context of NTDs, type 1 error is akin to undertreatment (failing to classify an area that is endemic or prematurely stopping MDA), while a type 2 error equates to overtreatment (providing treatment in an area that is non-endemic or no longer requires MDA). One can think of these parameters as levers that can be shifted up or down until the right balance of statistical rigor and feasibility is reached. Sticking with the example of the LF TAS, this survey was designed to have a 5% chance of type 1 error (i.e., 5% of the time, the TAS will falsely conclude that evaluation units with true prevalence above the threshold are safe to stop MDA) and 75% power when the true prevalence is 1% (i.e., 75% of the time, the TAS will correctly conclude that evaluation units that have achieved the threshold are safe to stop MDA) [3]. If one were to conduct the TAS using a tool that is 80% sensitive and 99% specific, then the actual type 1 error and power would be 0.0043% and 3% (see S1 Text). A survey with only 3% power to detect areas that have achieved success would be the death knell for any elimination program.

Fortunately, there are ways to improve decision-making performance when dealing with imperfect tests. For example, if a diagnostic test is <100% sensitive, one can account for this in the M&E survey by increasing the sample size, reducing the critical cutoff, or increasing our tolerance for committing a type 1 error. Similarly, if a diagnostic test is <100% specific, one can increase the critical cutoff or increase our tolerance for committing a type 2 error. The degree of modification to the M&E survey is dependent upon the accuracy of the test. Obtaining this information can be challenging for NTD practitioners who too often lack a gold standard against which to evaluate test performance. Well-characterized serum banks have been used to quantify test performance under ideal, laboratory-based conditions; however, moving forward, it is crucial that test performance be evaluated and summarized based on performance in the field under a representative range of epidemiologic settings. Such field trials are needed not only to understand test performance in different prevalence and co-endemicity settings, but equally important to assess the ease of test use and ensure that adequate test performance can be achieved in the hands of program teams. Finally, it is crucial that the data from both the laboratory and field validations be made available to WHO and the global community to enable appropriate modifications to the M&E surveys to maintain the decision-making integrity for NTD programs.

## Conclusions

While NTD programs have seen tremendous progress over the past decade in closing the mapping gap, scaling up MDA, and developing rigorous M&E frameworks, adequate consideration has not been paid to the diagnostic testing needs. The PC NTDs need new diagnostic tests explicitly designed to support stop MDA decisions and document the achievement of sustained elimination. Such tests will need to be highly specific to measure the low prevalence thresholds indicative of elimination. Just how specific will depend on the target threshold and sampling strategy employed by the M&E tool. If the specificity requirements are prohibitively high, test developers and program leaders should seek creative solutions. Combining tests serially, in the case of a confirmatory assay, or in parallel, by creating a multiplex test, can result in significant gains in net specificity. Finally, it is important to recognize that no test will be perfect, and it is imperative that M&E decision rules be adapted to reflect test performance in the field. Making these 4 important changes in the way that NTD diagnostics are designed and employed will result in tests that are better suited for making public health decisions in the context of NTD elimination.

## Supporting information

S1 Text R code for recreating the in-text examples. (DOC)
