# Supplementary material for: Time for a diagnostic sea-change: Rethinking neglected tropical disease diagnostics to achieve elimination
Source: PLoS Negl Trop Dis. 2020 Dec 31;14(12):e0008933. doi: 10.1371/journal.pntd.0008933 (PMC7774841; doi:10.1371/journal.pntd.0008933)
Supplement: S1 Text — (DOC) [file pntd.0008933.s001.doc]

**S1_Text.** R code for recreating the in-text examples.

# The probability of observing >x positive cases during a Transmission Assessment Survey

# with a sample size of n kids, a test that is Se sensitive and Sp specific and disease prevalence of prev.

#Example 1: Sample size = 1692, critical cutoff = 20, prevalence = 0%, Se = 100%, Sp = 99%

n <- 1692

x <- 20

prev <- 0

se <- 1

sp <- 0.99

test_pos <- se*prev + (1-sp)*(1-prev)

1-pbinom(x,n,test_pos)

#Example 2: Sample size = 1692, critical cutoff = 20, prevalence = 0.5%, Se = 100%, Sp = 99%

n <- 1692

x <- 20

prev <- 0.005

se <- 1

sp <- 0.99

test_pos <- se*prev + (1-sp)*(1-prev)

1-pbinom(x,n,test_pos)

#Example 3: Calculating Type 1 error: the probability of observing <=20 positives when the true #prevalence is 2%, assuming a sample size = 1692, Se = 80%, and Sp = 99%

n <- 1692

x <- 20

prev <- 0.02

se <- .8

sp <- 0.99

test_pos <- se*prev + (1-sp)*(1-prev)

Type_1_error = pbinom(x,n,test_pos)

Type_1_error

#Example 4: Calculating Power: the probability of observing <=20 positives when the true prevalence

# is 1%, assuming a sample size = 1692, Se = 80%, and Sp = 99%

n <- 1692

x <- 20

prev <- 0.01

se <- .8

sp <- 0.99

test_pos <- se*prev + (1-sp)*(1-prev)

Power = pbinom(x,n,test_pos)

Power
